# Mr. Carlisle's Case of Strangulated Hernia

**Published:** 1804-10-01

**Authors:** A. Carlisle

**Affiliations:** Soho Square


### To Dr. BATTY.

My dear Sir,

I Have sent you a Case for the Medical Journal, the publication of which may possibly tend to encourage doubtful endeavours on similar occasions, rather than abandon the unfortunate to certain death. The only cause to which I can attribute this extraordinary power of resisting a fatal disease, was the established habit of drinking spirituous liquors, the influence of which I have remarked, in other instances, to retard inflammatory terminations. The case may serve also to support the practice of operating for strangulated hernia, even in the most advanced stages, and under disagreeable symptoms.

I am, &c.

*Soho Square, Sept.* 3, 1804. A. CARLISLE.

---

ANN SPOONER, a corpulent woman, aged 56 years, for some time a resident in the Westminster Hospital, had an umbilical hernia, protruding about the bigness of half an ostrich's egg. The parietes of the tumour were thin, the contained viscera adhered to the sac, and the aperture in the abdomen was capable of admitting three fingers. She had several times suffered temporary strangulations, which had been relieved by bleeding from the system, and topical bleeding with leeches, &c. On the morning of the 28th of August last, as she drew up her stocking, a sudden protrusion took place, and the hernial tumour became painful and strangulated. The usual methods were employed, but at two o'clock the same day, the tumour burst its coverings, having become mortified, and a large portion of intestine, inflated, and of a livid hue, protruded into the bed. The distention of the intestine prevented its being returned; warm fomentations were applied, and opium with cordials administered. At ten o'clock in the evening I found her with the protruded intestine perfectly dead, and putrescent. A line of separation marked the two living ends, the intermediate mortified portion was found to be a continuous canal, and, as it afterwards proved, upwards of seven feet in length. The mesentery was alive within half an inch of the gut, a line of separation being visible, and the peritoneal coat both on that and the intestine was vesicated at the mortified border. The patient's strength and spirits were good; she could turn in bed, converse with coolness, and was attentive to ordinary wants and accommodations; her pulse was 115, her skin warm and perspirable, and she had no hiccup. I felt a strong desire to remove the mortified intestine, and to bring the two living ends of that canal together by ligature, over a roll of tough paste; but it was thought too hazardous for the small chance of good to be derived. On Wednesday, the 29th, she continued as before; the intestine became more putrid and more inflated: her pulse was regular, and from 105 to 120; she had occasional hiccup; her strength was by no means sunk. It was still judged imprudent to attempt any operation; she took wine and opium, and had a small alvine evacuation. The pulp of scraped carrot root was applied over the mortified parts, to diminish the putrescent effluvia. Without any remarkable change in the symptoms, in the appearance of the parts, she continued to become gradually weaker until four o'clock on Friday, the 31st of August, when she died.

On opening the body, the peritoneum and all the viscera contained within the abdomen were free from the marks of inflammation; the mortification of the bag which had contained the protruded intestine, was limited to the margin of the hernial aperture, which was about two inches in diameter. The previously adhering parts of the hernia had been all pushed out, and become disunited by the solution of the membranes. The sound border of the mesentery, and the two sound ends of the gut, were ulcerated on their surfaces, and covered with a purulent fluid. The mortified portion of gut proved to be the intestinum ileum, the dead part terminating within two inches of its entrance into the cæcum; it was measured, (after a partial dissection from the mesentery) and proved to be six feet three inches, although it was not nearly extended into a straight line.

The remarkable points of this case are, the long continuance of life and the limited injury sustained from such extent of disease. The practical inference to be deduced seems in favour of an operation such as that proposed; but it cannot be expected that many cases will occur, wherein the powers of life shall suffer so little, and the disease be so strictly confined.

